# Fast stabilization of a high-energy ultrafast OPA with adaptive lenses

**DOI:** 10.1038/s41598-018-32182-y

**Published:** 2018-09-25

**Authors:** Matteo Negro, Martino Quintavalla, Jacopo Mocci, Anna G. Ciriolo, Michele Devetta, Riccardo Muradore, Salvatore Stagira, Caterina Vozzi, Stefano Bonora

**Affiliations:** 1 CNR-IFN, P.zza Leonardo da Vinci 32, IT - 20133 Milano, Italy; 2 CNR-IFN, Via Trasea 7, IT - 35131 Padova, Italy; 3 Dipartimento di Informatica - Universitá di Verona, Strada Le Grazie 15, IT - 37134 Verona, Italy; 4 Dipartimento di Fisica - Politecnico di Milano, P.zza Leonardo da Vinci 32, IT - 20133 Milano, Italy

## Abstract

The use of fast closed-loop adaptive optics has improved the performance of optical systems since its first application. Here we demonstrate the amplitude and carrier-envelope phase stabilization of a high energy IR optical parametric amplifier devoted to Attosecond Science exploiting two high speed adaptive optical systems for the correction of static and dynamic instabilities. The exploitation of multi actuator adaptive lenses allowed for a minimal impact on the optical setup.

## Introduction

For a long time Ti:Sapphire amplified lasers, emitting around 800 nm, have been the only reliable sources of intense and ultrashort light pulses for numerous applications in Attosecond Science and strong-field Nonlinear Optics^1^. The last two decades have however witnessed an increased interest in Infrared Optical Parametric Amplifiers (IR-OPAs) as alternative sources for those applications^2–8^. OPAs have been extensively developed since the availability of intense pump lasers^9^ and exploited also in low-energy applications like ultrafast spectroscopy^10–12^; nowadays the scaling of these sources towards very high pulse energies by OPCPA^13^ and high repetition rates^14^ using fiber pump lasers is also achievable.

OPAs provide several benefits like huge spectral tunability and ultrashort pulse durations^15^, support of passive stabilization schemes for the Carrier-Envelope Phase (CEP)^16,17^ and access to large photon and photoelectron energies in strong optical field phenomena such as high-order harmonic generation^18^ and tunnel electron ionization^19^.

A limitation of IR-OPAs however comes from the requirement of a very good spatial quality and intensity stability of the pump beam that can hinder the scalability towards high pulse energies.

Furthermore, the performance of passive CEP stabilization schemes exploited in OPAs might be limited by the presence of phase-intensity noise coupling, that occurs whenever nonlinear optical processes are considered^20^; this again calls for very stable pumping lasers.

System stability can be increased using Adaptive Optics (AO).

For example, AO has been successfully used to increase the yield of non-linear phenomena exploiting iterative optimization algorithms^21–23^ or to correct for wavefront aberrations induced by uneven thermal dissipation^24–26^. In the latter cases, the aberrations were corrected using a wavefront sensor and a closed loop control operating at 10 Hz.

In some cases however, instability shows a fast time varying behavior, that would require astronomical grade adaptive optics for an effective correction. Such a system implies a consistent complexity due to the need for a fast wavefront modulator and closed loop control operating at few hundreds of Hz requiring specific hardware, such as Field Programmable Gate Arrays (FPGAs) or Graphics Processing Units (GPUs). A drawback of AO systems in general is due to the use of deformable mirrors as wavefront modulators that implies important modifications to existing laser optical setup.

Here we report on the first application of fast closed loop adaptive optics based on adaptive lenses for the fast stabilization of a home-built high energy IR parametric source pumped by a 1-kHz, 10-mJ, 25-fs Ti:Sapphire laser. In particular, we were able to implement two separate and independent closed loop control systems operating at the same time in different positions of the system. This was made possible thanks to a new approach consisting in the use of multi actuator adaptive lenses^27^ and a recently developed software package capable of working up to 500 Hz on a standard computer^28^. The closed loop control system was based on the use of a fast wavefront sensor (operating up to 500 Hz) triggered by the laser source. The multi actuator adaptive lens modulates the wavefront by changing its own shape. The lens is composed by two thin glass membranes and is filled with a BK7-dispersion-compatible liquid. Through the use of two ring shaped piezo electric actuators placed on the two faces of the lens, the wavefront can be modualted up to the 4th order of Zernike polynomials. The advantage of transmissive deformable optical elements is in the easiness of the installation that requires minimal changes to the existing optical setup^27^. While deformable mirrors can reach very high damage thresholds only with the use of dielectric coatings tuned on the laser wavelength, adaptive lenses can operate on intense laser beams over a broad spectral range (a discussion about lens transmission, working principle, chromatic aberration and damage threshold is reported in the section Adaptive Optics setup of the Supplementary Material).

**Figure 1:**
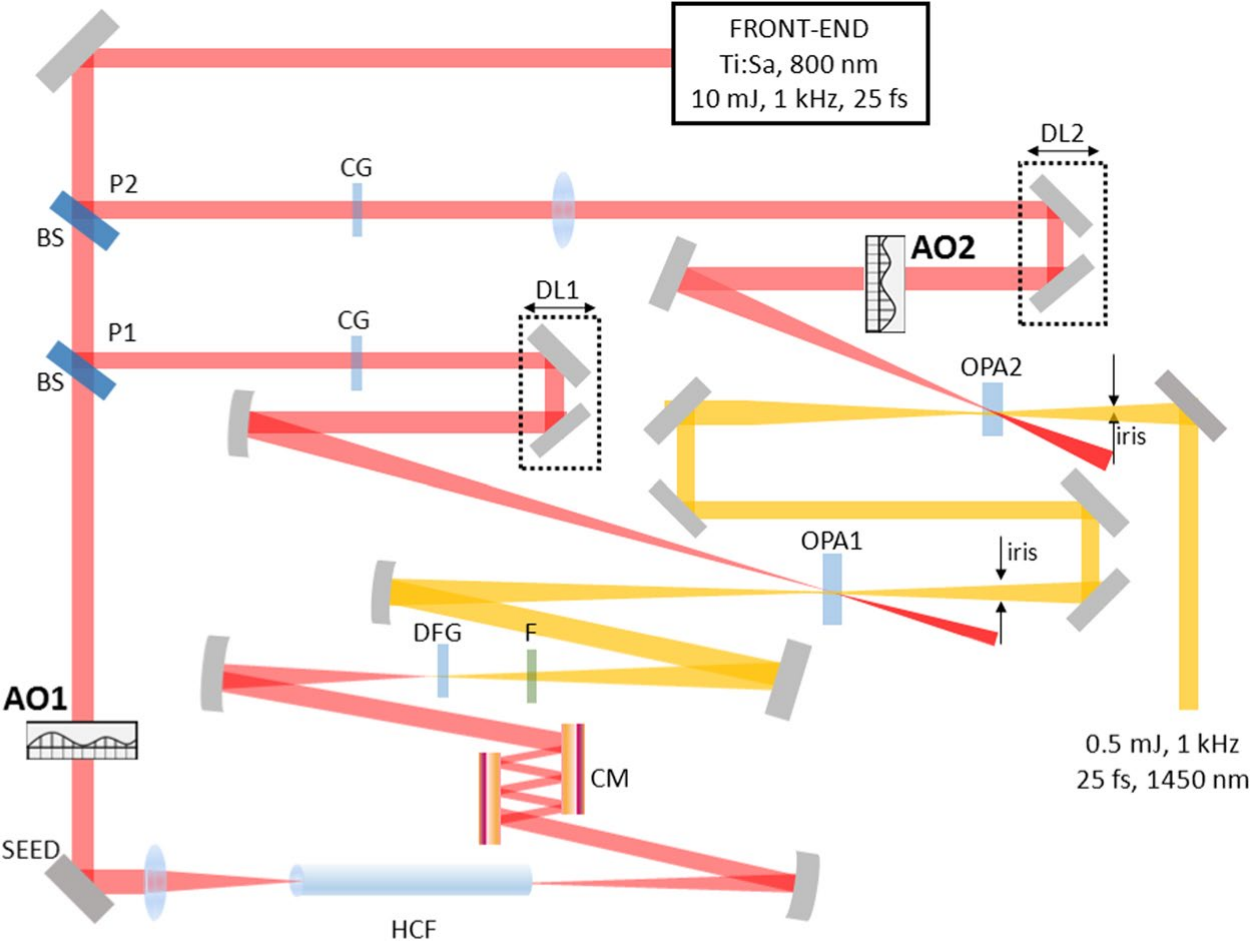
Experimental setup: AO1, AO2 adaptive optics; P1, P2 pump beams of OPA1 and OPA2 stages; DFG difference frequency generation; F filter; CM chirped mirrors; HCF hollow-core fiber; DL1, DL2 delay lines; CG compensation glass.

The experimental setup is shown in Fig. 1: a portion of the pump laser beam undergoes spectral broadening and compression down to 10 fs exploiting a hollow-core fiber compressor (HCF)^29^; an ultrashort IR seed is then obtained by intra-pulse difference frequency generation (DFG) driven by the compressed laser pulse in a BBO crystal. This scheme allows to generate an IR light pulse with a stable CEP^16^, which is a fundamental requirement for applications to the study of strong-optical-field phenomena in matter^1^. The seed is then amplified to the millijoule level in a two-stage OPA based on BBO crystals in type II phase-matching, that are pumped by the remaining fraction of the laser pump beam^18^. Since the overall OPA layout spans about 10 m, it is subject to environment instabilities such as temperature fluctuations, air fluxes etc.

We implemented two AO systems in the OPA layout: the first one (AO1) was placed in the collimated laser beam before the focusing section of the HCF, whereas the second AO system (AO2) was placed on the pump beam driving the second OPA stage (see Fig. 1). Each AO system is based on a Hartmann-Shack wavefront sensor (WFS) and a multi-actuator deformable lens^27^ with 10-mm clear aperture, allowing to correct aberrations of the trasmitted laser beam up to the 4th order of Zernike polynomials. It is worth noting that nonlinear optical effects in the lenses, that might lead to phase front aberrations, are in this way corrected as well. The WFSs are optically conjugated to the deformable lenses by means of two lenses telescopes and analyze a tiny fraction of the beam that is extracted by exploiting the loss of two mirrors (see Fig. S1 of the Supplementary Material). The adaptive lenses used in this paper did not report any damage after two weeks of continuous operation (see again the Supplementary Material for the damage threshold characterization).

## Results and Discussion

**Figure 2:**
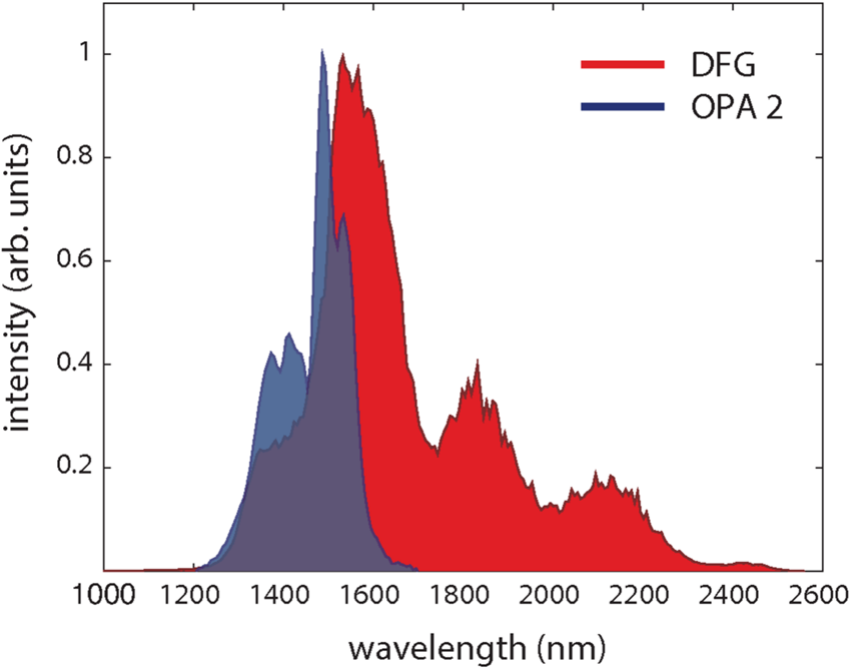
Single-shot normalized spectra of the IR seed pulse produced by difference frequency generation (red) and of the output of the second stage of the IR-OPA (blue).

**Figure 3:**
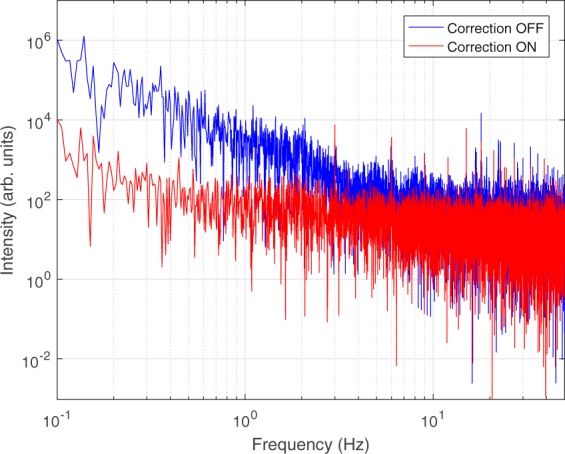
Power spectral density of the integrated IR seed spectrum without (blue curve) and with (red curve) correction actuated with system AO1.

Figure 2 shows the IR seed spectrum (red pattern) acquired by an infrared spectrometer as generated by DFG at the front end of the OPA. In a first set of measurements we characterized the stability of this seed by measuring the integral of its single-shot spectrum with an acquisition rate limited by the hardware to about 100 Hz; the time-series was then analyzed in the Fourier domain. Figure 3 shows the power spectral density of the acquired signal (blue curve) without any correction on the laser beam. The power spectrum shows significant noise components up to few Hz. By turning the AO1 system on, a significant improvement on the seed stability is achieved (red curve). A close inspection of the figure reveals some deterministic noise components at 3 Hz and its harmonics, that are related to the mechanical vibrations transferred from the cryogenic compressor mounted on the Ti:Sapphire power amplifier to the optical bench. A reduction of the IR seed fluctuations can be observed up to tens of Hz, although with less efficiency at high frequencies with respect to the low ones. However, the peaks of the deterministic noise components appear unaffected by the AO correction.

**Figure 4:**
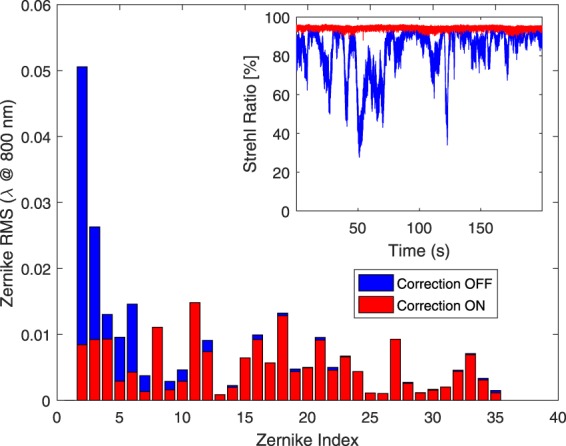
Standard deviation of the fluctuation of the Zernike terms (1: piston, not shown; 2,3: tip/tilt; 4: defocus; 5–6: astigmatism) without (blue histogram) and with (red histogram) the correction actuated by system AO1. Inset: Strehl ratio evolution at the HCF input calculated in the two cases.

The analysis of the correction terms introduced by the AO1 system is reported in Fig. 4, where we show the standard deviation of the Zernike terms without (blue) and with (red) the AO1 system in operation. We observed a major influence of the environmental conditions on the first 5 Zernike terms that are the ones relative to the pointing stability (tip, tilt), defocus and astigmatism (0 and 45 degrees). The whole correction provided by the AO1 system improves considerably both the stability and the quality of the laser beam coupling to the HCF, as shown by the inset of Fig. 4 that displays the temporal evolution of the Strehl ratio of the laser beam (i.e. the ratio between the peak intensity of an aberrated wavefront and that of a perfect wavefront in the focal plane) determined at the HCF input over a period of 180 s. In particular, the Strehl ratio becomes very stable when the AO1 system is operating (red line), whereas shows large variations when no correction is applied (blue line). On the average, this parameter improves from 83.5% to 94.7%. This improves also the stability of the spectrum of the compressed laser pulses, that is transferred to the IR seed produced by DFG between the spectral tails of those pulses.

In a second set of measurements we performed a characterization of the performance of the IR-OPA source by exploiting both AO systems. In all the measurements we tuned the OPA around 1.45 *μ*m by suitable setting of the BBO crystals orientation; Fig. 2 shows the corresponding pulse spectrum at the output of the OPA (blue pattern). In this condition the energy of the output pulses was about 500 *μ*J and the pulse duration, measured by a Frequency-Resolved Optical Gating (FROG) system, was about 25 fs. Even in this case, the stability of the source was evaluated by measuring the integral of the single-shot output spectrum at a 100-Hz acquisition rate.

**Figure 5:**
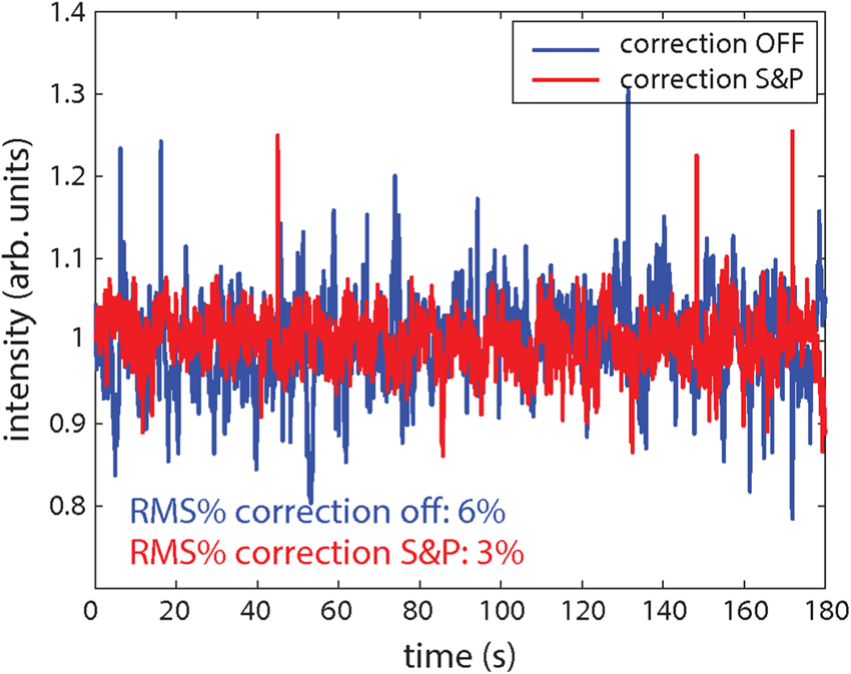
Evolution of the integral of the output OPA spectrum as a function of time without (blue curve) and with (red curve) correction actuated with both system AO1 and AO2 operating on the OPA seed (S) and pump (P) branches.

Figure 5 shows the evolution of the OPA output measured over 180 s; when the two AO systems are not operating, the r.m.s. fluctuation of the signal amounts to about 6% (blue curve); this fluctuation is halved by turning on both AO1 and AO2 (red curve). Both AO systems contribute to the improvement of the OPA stability; indeed the r.m.s. signal fluctuation increases to 4% when only AO1 is operating (Fig. 8 in the Supplementary Material), thus indicating an important role of the pump beam instability on the performaces of the OPA. The power spectral density of the OPA signal provides features similar to those observed in the seed case; in particular a significant improvement is observed on the slow noise components, whereas the correction becomes less effective at higher frequencies. It is worth pointing out that the AO systems can correct for beam aberrations, but are ineffective on fluctuations of the laser pulse energy; those fluctuation are responsible for the residual instability of the OPA output energy observed in Fig. 5.

A detailed investigation was also performed on the stability of the Carrier-Envelope Phase of the amplified pulses, that turned out to be affected by the instabilities of the pumping laser. The characterization of the CEP fluctuation was performed using a standard f-2f nonlinear interferometer coupled to a near-infrared spectrometer and based on white light generation in a thin fused silica plate followed by second harmonic generation in a BBO crystal^16^. In our experimental setup this CEP instability may have several sources: the first one comes from an instability in the coupling between the laser beam and the HCF, which translates in fluctuations in energy, spectral bandwidth and spectral phase of the compressed laser pulses at the HCF output. This noise is then transferred to the CEP of the seed IR pulses produced by DFG. A second noise source may come from the OPA stages, where the instability of pointing direction and intensity of the pump pulse may be translated to phase noise on the amplified seed by third order nonlinear effects^20^. As a last point, one has to consider that additional noise could be introduced by the instrumentation exploited for the CEP characterization, since f-2f interferometers are also prone to phase-intensity noise coupling^30^. In all those cases, the CEP fluctuations can be considerably reduced by improving the stability of the pump source.

**Figure 6:**
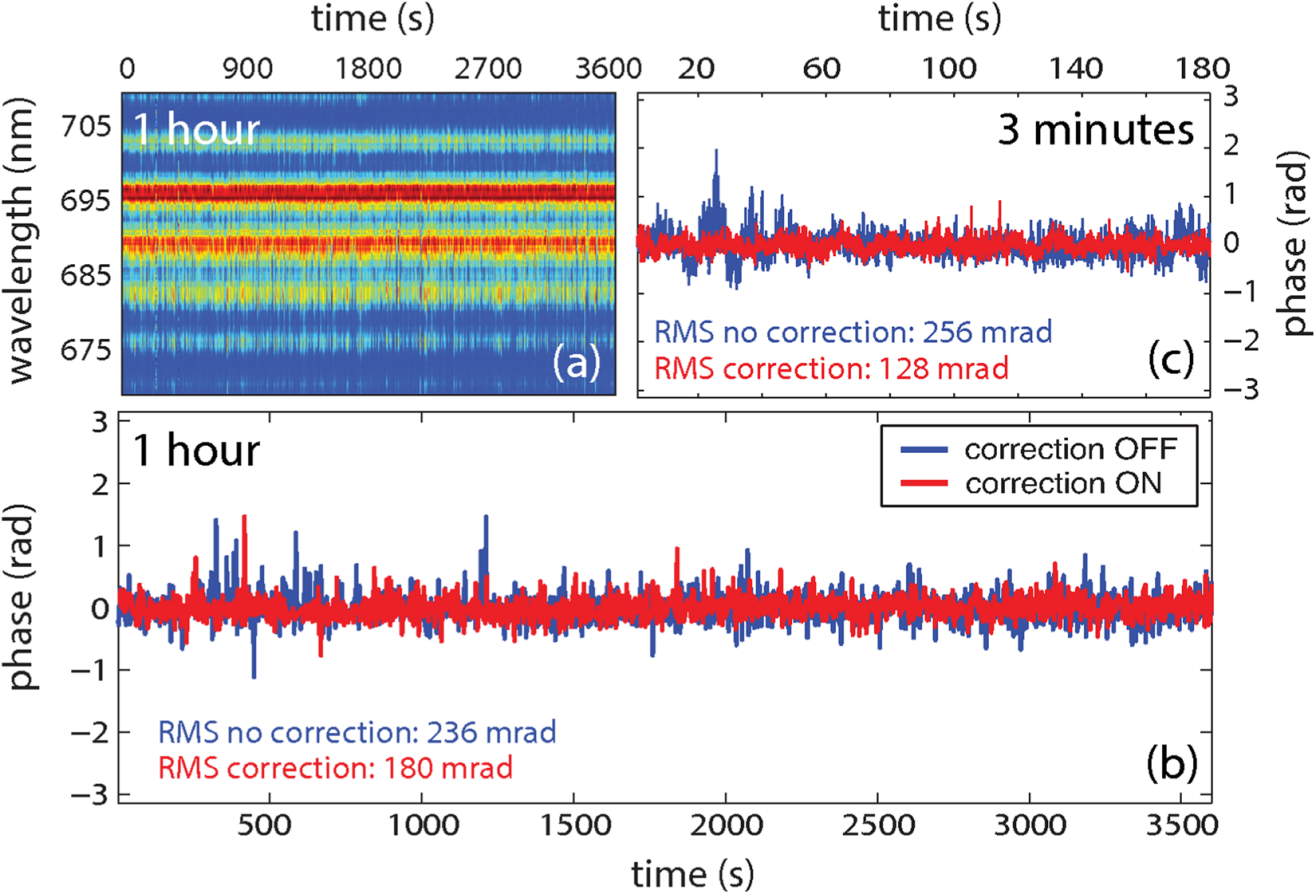
Characterization of the CEP fluctuations at the IR-OPA output: (**a**) scan of the spectral interference pattern acquired by the f-2f interferometer over 1 hour when the AO systems were operating. (**b**,**c**) Retrieved CEP evolution without (blue curve) and with correction operated by the AO1 and AO2 systems (red curve) over 1 hour (**b**) and over a period of 3 minutes (**c**).

Figure 6(a) shows a scan of the spectral interference pattern acquired by the f-2f interferometer over 1 hour when both AO systems were operating; a similar scan was acquired when the two AO systems were not operating. The retrieved CEP evolution, determined from those scans by Fourier analysis, is reported in Fig. 6(b,c) in the cases without (blue curve) and with (red curve) the correction operated by the AO1 and AO2 systems. As shown in Fig. 6(c) the analysis restricted to a period of time of 3 minutes displays a considerable reduction of the CEP instability operated by the AO correction, with a decrease of the corresponding r.m.s. value from 256 mrad to 128 mrad. This improvement is still significant over the whole 1-hour acquired scan, corresponding to a decrease of the r.m.s. value from 236 mrad to 180 mrad. This noticeable result boosts the CEP stability performaces to a level comparable to active stabilization systems operating in standard high-energy Ti:Sapphire sources.

## Conclusions

We reported on the first exploitation of deformable lenses in the stabilization of a high-energy IR parametric source designed for Attosecond Science applications. The combined use of adaptive lenses, in the place of deformable mirrors, and fast wavefront sensors allowed us an easy implementation of two adaptive optics systems operating at the same time on a high-energy laser source without any change to the optical setup. With respect to similar experiments reported in literature we have been able to correct for dynamic aberrations (sampling frequency up to 500 Hz) and to implement two independent aberration correction systems, placed in the most sensitive positions of the laser. The low absorption of the deformable lens allows to correct aberrations of mJ-level femtosecond pump beams, while the fast response of the AO system ensures the tracking of common instabilities in laser sources. In particular, beam pointing fluctuation and wavefront aberration induced by air turbulence and thermal instabilities can be promptly corrected. The significant improvement introduced by this AO system on the IR-OPA performances, in terms of both output energy and pulse CEP stability, is a further motivation towards the reliable long-term exploitation of high-energy few-cycle parametric sources in strong-optical-field Physics.

## Electronic supplementary material

Supplementary material

## Data Availability

Data analysed during the current study are available from the corresponding author on reasonable request.

## References

[1] Krausz F, Ivanov M (2009). Attosecond physics. Rev. Mod. Phys..

[2] Shan B, Chang Z (2001). Dramatic extension of the high-order harmonic cutoff by using a long-wavelength driving field. Phys. Rev. A.

[3] Xu H (2008). Fine interference fringes formed in high-order harmonic spectra generated by infrared driving laser pulses. Phys. Rev. A.

[4] Colosimo P (2008). Scaling strong-field interactions towards the classical limit. Nature Physics.

[5] Takahashi EJ, Kanai T, Nabekawa Y, Midorikawa K (2008). 10 mJ class femtosecond optical parametric amplifier for generating soft x-ray harmonics. Applied Physics Letters.

[6] Vozzi C (2010). High harmonic generation spectroscopy of hydrocarbons. Applied Physics Letters.

[7] Vozzi C, Negro M, Stagira S (2012). Strong-field phenomena driven by mid-infrared ultrafast sources. Journal of Modern Optics.

[8] Thiré N (2015). 10 mJ 5-cycle pulses at 1.8 m through optical parametric amplification. Applied Physics Letters.

[9] Dubietis A, Jonušauskas G, Piskarskas A (1992). Powerful femtosecond pulse generation by chirped and stretched pulse parametric amplification in BBO crystal. Optics Communications.

[10] Nisoli M (1998). Parametric generation of high-energy 14.5-fs light pulses at 1.5-micron. Opt. Lett..

[11] Cerullo G, Nisoli M, Stagira S, Silvestri SD (1998). Sub-8-fs pulses from an ultrabroadband optical parametric amplifier in the visible. Opt. Lett..

[12] Riedle E (2000). Generation of 10 to 50 fs pulses tunable through all of the visible and the NIR. Applied Physics B.

[13] Witte S, Eikema KSE (2012). Ultrafast Optical Parametric Chirped-Pulse Amplification. IEEE Journal of Selected Topics in Quantum Electronics.

[14] Andersen TV (2006). High repetition rate tunable femtosecond pulses and broadband amplification from fiber laser pumped parametric amplifier. Opt. Express.

[15] Cerullo G, De Silvestri S (2003). Ultrafast optical parametric amplifiers. Rev. Sci. Instrum..

[16] Vozzi C (2007). Millijoule-level phase-stabilized few-optical-cycle infrared parametric source. Opt. Lett..

[17] Cerullo G, Baltuska A, Muecke OD, Vozzi C (2011). Few-optical-cycle light pulses with passive carrier-envelope phase stabilization. Laser & Photonics Reviews.

[18] Ciriolo AG (2017). Optical Parametric Amplification Techniques for the Generation of High-Energy Few-Optical-Cycles IR Pulses for Strong Field Applications. Applied Sciences-Basel.

[19] Popmintchev T (2012). Bright Coherent Ultrahigh Harmonics in the keV X-ray Regime from Mid-Infrared Femtosecond Lasers. Science.

[20] Baltuška A (2003). Phase-controlled amplification of few-cycle laser pulses. IEEE Journal of Selected Topics in Quantum Electronics.

[21] Bartels R (2000). Shaped-pulse optimization of coherent emission of high-harmonic soft X-rays. Nature.

[22] Villoresi P (2004). Optimization of high-order harmonic generation by adaptive control of a sub-10-fs pulse wave front. Opt. Lett..

[23] Bonora S (2012). Optimization of low-order harmonic generation by exploitation of a resistive deformable mirror. Applied Physics B.

[24] Bonora S, Pilar J, Lucianetti A, Mocek T (2016). Design of deformable mirrors for high power lasers. High Power Laser Science and Engineering.

[25] Novák O (2015). Status of the High Average Power Diode-Pumped Solid State Laser Development at HiLASE. Applied Sciences.

[26] Fourmaux S (2008). Laser beam wavefront correction for ultra high intensities with the 200 TW laser system at the Advanced Laser Light Source. Opt. Express.

[27] Bonora S (2015). Wavefront correction and high-resolution *in vivo* OCT imaging with an objective integrated multi-actuator adaptive lens. Opt. Express.

[28] Mocci, J., Quintavalla, M., Trestino, C., Bonora, S. & Muradore, R. A multi-platform CPU-based architecture for cost-effective adaptive optics systems. *IEEE Transactions on Industrial Informatics*, 10.1109/TII.2018.2799874 (2018).

[29] Cerullo G (2000). Few-optical-cycle laser pulses: From high peak power to frequency tunability. IEEE Journal of Selected Topics in Quantum Electronics.

[30] Li C (2007). Determining the phase-energy coupling coefficient in carrier-envelope phase measurements. Opt. Lett..

