# Supplementary material for: Fast stabilization of a high-energy ultrafast OPA with adaptive lenses
Source: Sci Rep. 2018 Sep 25;8:14317. doi: 10.1038/s41598-018-32182-y (PMC6156574; doi:10.1038/s41598-018-32182-y)
Supplement: Supplementary file 1 — Supplementary material [file 41598_2018_32182_MOESM1_ESM.pdf]

Fast stabilization of a high-energy ultrafast OPA with adaptive lenses: supplementary material

Matteo Negro[1], Martino Quintavalla[2], Jacopo Mocci[3], Anna G. Ciriolo[4], Michele Devetta[1], Riccardo Muradore[3], Salvatore Stagira[4,1,*], Caterina Vozzi[1], and Stefano Bonora[2]

[1]CNR-IFN, P.zza Leonardo da Vinci 32, IT - 20133 Milano, Italy
[2]CNR-IFN, Via Trasea 7, IT - 35131 Padova, Italy
[3]Dipartimento di Informatica - Universitá di Verona, Strada Le Grazie 15, IT - 37134 Verona, Italy
[4]Dipartimento di Fisica - Politecnico di Milano, P.zza Leonardo da Vinci 32, IT - 20133 Milano, Italy
[*]salvatore.stagira@polimi.it

ABSTRACT

This document provides supplementary information to “Fast stabilization of a high-energy ultrafast OPA with adaptive lenses”. The optical setup used for Adaptive optics (AO) correction of the seed and pump beams on a high-energy optical parametric amplifier is described; measurement not reported in the main text that were performed on the laser beam wavefront and on the stability of the OPA source are presented here.

Adaptive optics setup

Figure 1 reports in detail the optical layout of the two AO systems operating on (a) the beam focused in the Hollow Core Fiber (HCF beam in the following) and (b) the OPA2 pump beam; see Fig. 1 of the main text for the scheme of the OPA source. In both cases, we used the leakage of the back surface of a high reflectivity mirror to feed a Shack-Hartmann Wavefront Sensor (WFS) without any change to the original laser beam path. The lenses F1 and F2 were used to optically conjugate the

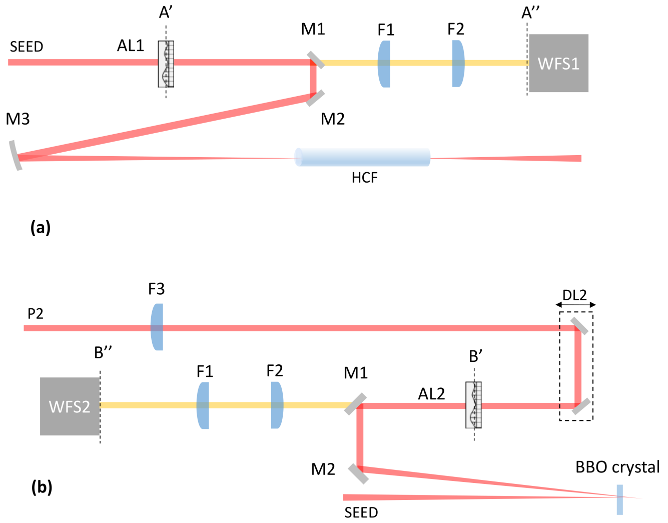

Figure 1. Optical setup of the AO systems operating on (a) the HCF beam and on (b) the OPA2 pump beam. AL1, AL2 multi actuator adaptive lenses; M1, M2 high reflectivity flat mirrors; M3 focusing spherical mirror; HCF hollow core fiber; DL2 delay line; F1,F2 beam reducing telescope lenses; F3 focusing lens; WFS1/2 wavefront sensors; A’, A”, B’, B” optically conjugate planes.

deformable lens to the WFS and to reduce the beam size to make it suitable for the WFS aperture. Focal lengths on the HCF beam were 75 and 25 mm for F1 and F2 respectively, providing a beam reduction from 10 mm to 3.33 mm on WFS1. Focal lengths on the OPA2 pump beam were 300 and 75 mm respectively, providing a reduction from 10 mm to 2.5 mm on WFS2. The transmission spectrum of the multi actuator adaptive lenses is approximately 90% from 400 to 1000 nm as reported in

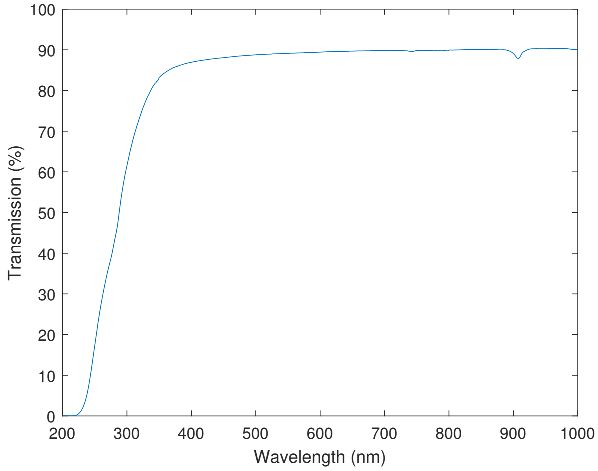

Figure 2. Transmission of the adaptive lens in the spectral range from 200 to 1000 nm.

Fig. 2. The transmission curve does not show any absorption peak in the region around 800 nm and could be further increased by using anti reflection coatings on the surface of the lens. The damage threshold of the adaptive lenses was determined by exposing a sample lens to increasing laser intensities until a permanent damage was achieved. We determined in this way a threshold at 25-fs pulse duration of 12.6 mJ/cm^2, that is about 4 times higher than the intensities to which the lenses were exposed during the experiment. It is worth noting that the damage threshold increases to 1.8 J/cm^2 for uncompressed laser pulses, thus demonstrating that these lenses are very suitable for the correction of high-power laser beams before compression. We evaluated also the chromatic aberration introduced by the lens because of the dispersion of the filling liquid. In the worst case condition of the deformable lens to its maximum deformation (equivalent to a lens of 1 m focal lentgth) the wavefront difference at the extremes of a spectrum of 800nm+/-40nm is below 0.02 waves rms, well below the Marechal criterion of well corrected optical systems (0.08 waves rms). The group delay dispersion introduced by the lens amounts to about 150 fs^2 at 800 nm; this dispersion can be easily precompensated either by chirped mirrors or by the compressor settings in amplified laser systems.

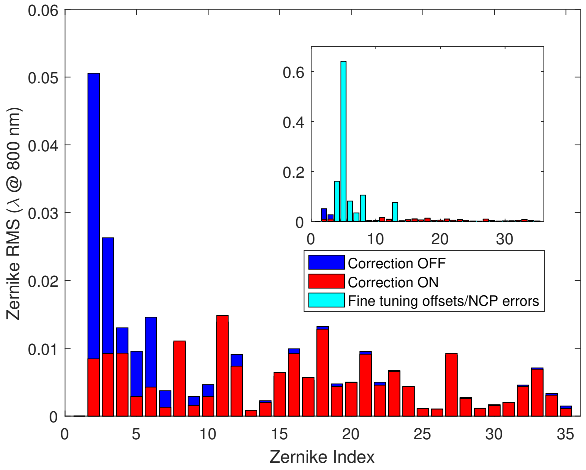

Figure 3. Zernike polynomials RMS coefficients recorded on the HCF optical beam without (blue) and with (red) AO correction. Inset: particular of the fine tuning offsets and NCP errors.

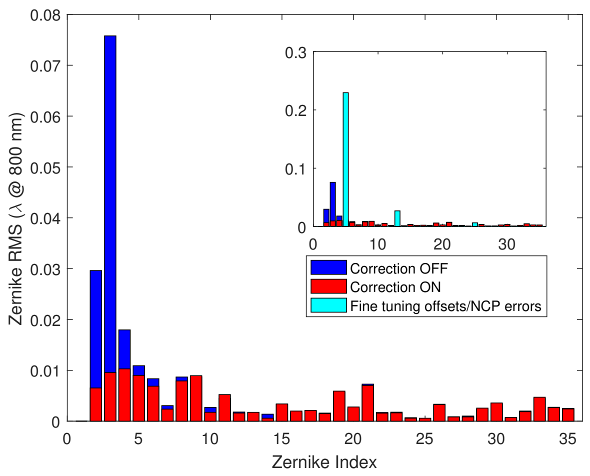

Figure 4. Zernike polynomials RMS coefficients recorded on the OPA2 pump optical beam without (blue) and with (red) AO correction. Inset: particular of the fine tuning offsets and NCP errors.

Adaptive Optics measurements

AO correction was performed by "Photon Loop" closed-loop control software (details can be found in[1]) developed to work with AO systems at 500 Hz on standard laptops using simple USB interfaces. Synchronization between the laser pulses and the data acquisition was provided by triggering the WFS cameras to the laser trigger signal. AO measurements consisted in the acquisition of the wavefront measurements for 200 s with and without AO correction.

In all the cases, when the adaptive optics was turned off, the adaptive lens was still inserted in the optical path, but was maintained in a flat, optically neutral shape. Aside from turbulence and vibration correction, the AO system also allowed for the correction of Non Common Path (NCP) errors induced by telescope lenses and by the optical elements introduced after the deformable lens including the last focusing optical element. We used the Photon Loop independent Zernike-control feature to reduce the non-common path error aberrations by maximizing the signal exiting the HCF while fine-tuning independently the value of each Zernike polynomial. In the case of the system on the pump beam of OPA2, the correction of the NCP was obtained by maximizing the signal of the IR amplified beam. The temporal RMS values of Zernike coefficients determined from 200 s time series without and with the AO correction as well as NCP errors and fine tuning offsets are reported in Fig. 3 for the HCF beam and in Fig. 4 for the OPA2 pump beam respectively.

The Strehl ratio, i.e. the ratio of the peak of the light intensity in the origin of the focal plane with respect to that of an ideal flat wavefront, was computed from the Zernike polynomials coefficients according to[2]:

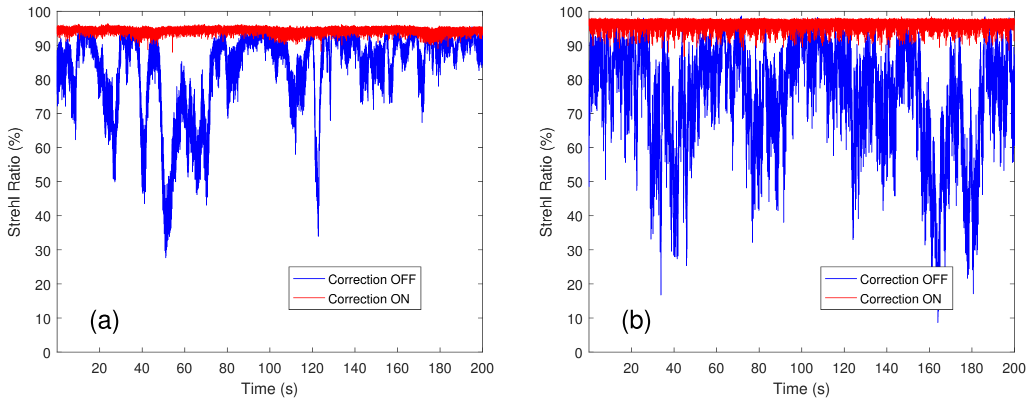

Figure 5. Strehl ratio calculated from WF measurement without (blue) and with (red) the AO correction on (a) the HCF beam and (b) the OPA2 pump beam.

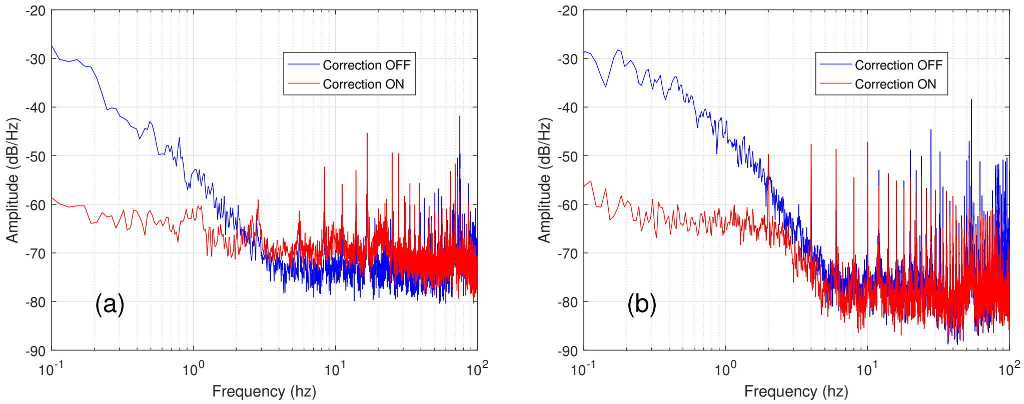

Figure 6. Power spectral density of the RMS deviation of the WF without (blue) and with (red) the AO correction for: (a) the OPA2 pump beam; (b) the HCF beam.

$$Strehl = e^{-\sigma^2} \tag{1}$$

where σ is the spatial variance of the WF phase and can be calculated from Zernike polynomials coefficients Z_n as[3]:

$$\sigma^2 = \sum_n \left(\frac{2\pi Z_n}{\lambda} \right)^2 \tag{2}$$

The Strehl ratio determined over 200 s for the HCF and the OPA2 pump beams is reported in Figure 5. The increase in the time averaged Strehl ratio after the correction is from 83.5% to 94.7% and from 76.3% to 97.0% respectively. Frequency domain analyses of the spatial RMS deviation of the WF allowed to evaluate the frequency response and effectiveness of the AO system. Power spectra for HCF and OPA2 pump beams without and with AO correction are reported in Fig. 6. The power spectral density plots highlight the presence of disturbances up about 10 Hz. Continuous spectra in the uncorrected measurements indicate turbulence induced aberration, while narrow spectral lines correspond to aberrations induced by mechanical vibrations of the cryogenic compressor of the Ti:sapphire power amplifier that are present at higher frequencies.

Stability at the DFG stage output

IR spectra collected after the difference frequency generation (DFG) stage and after amplification in the OPA2 stage were analyzed to retrieve the performance achieved with the introduction of AO correction. The evaluation of the stability of the IR seed produced by DFG was performed by evaluating the spectrum integral over 180 s, as shown in Fig. 7, and calculating its power spectral density that is reported in Fig. 3 of the main text.

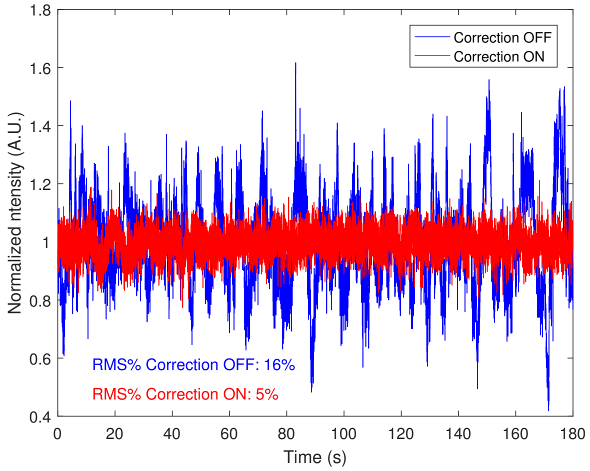

Figure 7. Measurement of the stability of the IR seed generated by DFG without (blue) and with (red) the AO correction during a 180 s scan.

Table 1. Configuration of the AO correction during the measurements on the IR spectra after amplification in the OPA2 stage.

AO configuration		
HCF arm	OPA2 pump arm	Measurement mode
off	off	AO OFF
on	off	AO Seed
off	on	AO Pump
on	on	AO ON

Stability at the OPA2 output

Evaluation of the IR signal stability after amplification in the OPA2 stage was performed considering the effect of AO correction both on the HCF and the OPA2 pump arms, hence taking into account four different cases, as listed in Table 1. Measurements of the signal stability in these four cases are reported in Fig. 8. The Carrier Envelope Phase (CEP) stability of the amplified IR pulses was also evaluated in these four cases by means of a f-2f interferometer and the phase was retrieved by tracking the interferometric fringes. The phase trend over 180 s measurements is reported in Fig. 9.

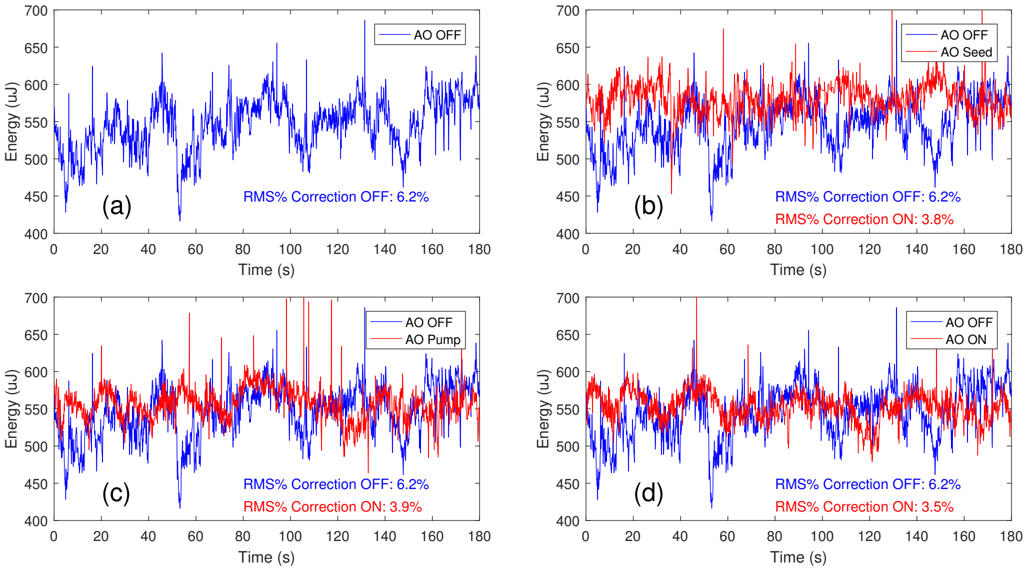

Figure 8. Measurement of the IR signal stability after amplification in the OPA2 stage for the four cases listed in Table 1.

Long-term measurements

AO and spectral measurements were carried out continuously over one hour to prove that the stabilization is reliable over long periods. WF stability measurements on both HCF and OPA2 pump arms yielded an increase on the averaged Strehl ratio after the correction from 82.1% to 95.6% and from 68.6% to 96.4% respectively. Corresponding long-term CEP stability measurements for the amplified IR pulses are shown in Fig. 6 of the main text.

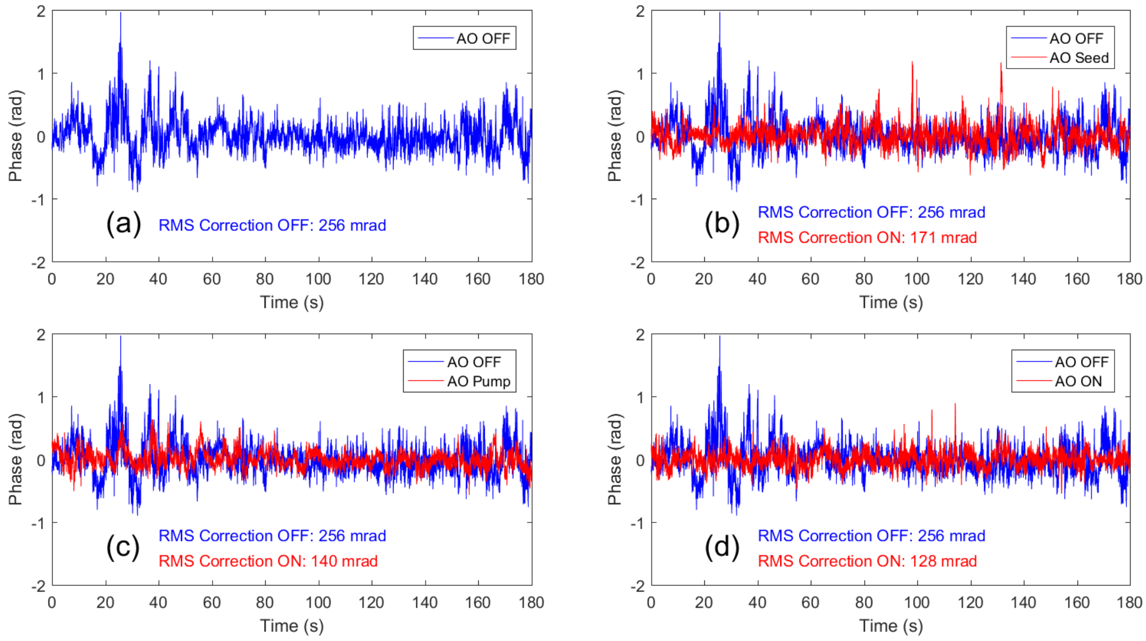

Figure 9. Measurement of the CEP phase stability of the IR pulses after amplification in the OPA2 stage for the four cases listed in Table 1.

References

1. Mocci, J., Bonora, S. & Muradore, R. Development of a CPU-based architecture for high performance adaptive optics systems. In Bonora, S. (ed.) *Proceedings of the 10th international Workshop on Adaptive Optics for Industry and Medicine*, 233 (CLEUP, 2015).
2. Tyson, R. *Introduction to adaptive optics* (SPIE, 2000).
3. Mahajan, V. N. Zernike Polynomial and Wavefront Fitting. In Malacara, D. (ed.) *Optical Shop Testing, 3rd Edition*, 507 (Wiley, 2007).
